# Effects of a Two-Month Exercise Training Program on Concurrent Non-Opiate Substance Use in Opioid-Dependent Patients during Substitution Treatment

**DOI:** 10.3390/jcm13040941

**Published:** 2024-02-06

**Authors:** Alexandros Psarianos, Costas Chryssanthopoulos, Athanasios Theocharis, Thomas Paparrigopoulos, Anastassios Philippou

**Affiliations:** 1 1st Department of Psychiatry, Medical School, National and Kapodistrian University of Athens, 11528 Athens, Greece; apsarianos@med.uoa.gr (A.P.); tpaparrig@med.uoa.gr (T.P.); 2 Greek Organization Against Drugs (OΚAΝA), 10433 Athens, Greece; ath.theocharis@gmail.com; 3 Department of Physiology, Medical School, National and Kapodistrian University of Athens, 11527 Athens, Greece; chryssan@phed.uoa.gr

**Keywords:** aerobic exercise, exercise training, non-opiate substance abuse, concurrent use, opioid users, medication for opioid use disorders

## Abstract

Background: This randomized controlled trial aimed to evaluate the effects of a two-month exercise intervention on the concurrent non-opiate substance use (alcohol, cocaine, cannabis, and benzodiazepines) in opioid users during their medication treatment. Methods: Ninety opioid users (41 females) in methadone and buprenorphine medication treatment were randomly divided into four groups: (a) buprenorphine experimental (BEX; n = 26, aged 41.9 ± 6.1 yrs); (b) buprenorphine control (BCON; n = 25, aged 41.9 ± 5.6 yrs); (c) methadone experimental (MEX; n = 20, aged 46.7 ± 6.6 yrs); and (d) methadone control (MCON; n = 19, aged 46.1 ± 7.5 yrs). The experimental groups (BEX and MEX) followed an aerobic exercise training program on a treadmill for 20 min at 70% HR_max_, 3 days/week for 8 weeks. Socio-demographic, anthropometric, and clinical characteristics, as well as non-opioid drug use in days and quantity per week, were assessed before and after the intervention period. Results: Following the exercise training, the weekly non-opioid substance consumption (days) decreased (*p* < 0.05) in both exercise groups and was lower in BEX compared to MEX, while no differences were observed (*p* > 0.05) between the control groups (BCON vs. MCON) or compared to their baseline levels. Similarly, the daily amount of non-opiate substance intake was reduced (*p* < 0.05) post-training in BEX and MEX, whereas it did not differ (*p* > 0.05) in BCON and MCON compared to the baseline. Conclusions: The two-month exercise intervention reduced the non-opioid drug use in both the methadone and buprenorphine substitution groups compared to the controls, suggesting that aerobic exercise training may be an effective strategy for treating patients with OUDs.

## 1. Introduction

Non-opioid polysubstance abuse is common among patients with OUDs seeking treatment [1]. It is usually observed during the early phases of medication for opioid use disorders and is enhanced by the experience of withdrawal and craving [2,3,4,5]. Specifically, the rates of this pattern of use are high (~65%) in patients receiving methadone or buprenorphine treatment, which is of concern due to its potential complications [6]. Such concerns are associated with the interaction of non-opioid substances with the treatment medication and the risk that may act as substitutes. Specifically, they potentially lead to increased (non-opioid) use, while they might also escalate the risk of substitution-substance use [7,8,9]. Consequently, the effect of drug therapy is compromised, and the risk of relapse increases [10].

The most common non-opioid abuse substances used by patients with OUDs are alcohol [11,12,13], benzodiazepines [14,15], cannabis [16], and cocaine [3,11,17]. Specifically, the most common patterns of this parallel use, recorded among patients undergoing methadone and buprenorphine replacement therapy in Europe, are (a) cannabis as the main substance, consumed with alcohol and cocaine; (b) benzodiazepines as the main substance, combined with cannabis and cocaine; and (c) cocaine as the main substance, consumed with cannabis and alcohol [18]. Interestingly, specific combinations, such as cocaine and benzodiazepines, can lead to increased opiate use during treatment or even to relapse and the abandonment of treatment [19]. Moreover, the combined use of cocaine and alcohol is particularly dangerous due to the increased risk of cardiovascular events. In addition, a dysfunctional behavior, mainly associated with a reduced judgment of patients for treatment, and a vulnerability to psychiatric disorders, such as depression, anxiety, and personality disorders, are also possible adverse effects of the combined use of cocaine and alcohol [20,21,22]. Moreover, the continuous and repeated use of addictive substances may cause adaptive, morphological, and functional alterations in the brain’s reward system and also in the system that selects the behaviors to satisfy the individual’s drives and desires, eventually forming an endophenotype vulnerable to both addiction and other risky behaviors [23,24,25,26].

Therefore, normally, a polysubstance use environment is formed beyond the substitute substance [18], which is associated with an increased risk of psychiatric and physical health problems [19]. Moreover, this polysubstance use results in significant impairments in social and cognitive function [6,27], affecting the patients’ judgment and behaviors [1]. In addition, it is consistently associated with worse treatment outcomes, including poor treatment maintenance, high relapse rates, and threefold increases in the mortality rate compared to that of the substitute substance use alone [28,29].

Physical exercise has become an increasingly popular, alternative therapeutic intervention for substance-dependent patients due to its high effectiveness and relatively low cost [30,31,32]. Indeed, a targeted exercise training program of adequate dose can improve energy levels [33], strengthen skeletal muscles [34], lower blood pressure and blood lipids [35], increase bone density [36], and regulate psychological parameters such as perceived anxiety, depressive symptoms, and stress levels [25,26]. Exercise may also reduce substance abuse, enhancing adherence to treatment in patients with cocaine [37], cannabis (Tetrahydrocannabinol—TCH) [38], alcohol [39], and benzodiazepines use disorders [40].

More specifically, exercise appears to be an ideal complementary strategy and, together with psychotherapeutic interventions, can have a positive effect on abstinence from alcohol use [39]. In particular, aerobic exercise can restore synaptic deficits caused by cocaine, thereby reducing drug-seeking behavior [37]. Moreover, physical exercise is a powerful activator of the endocannabinoid system, and, thus, it could be a very effective intervention for stopping cannabis use, reducing psychosomatic withdrawal, regulating stress management, and mitigating the desire for drug use [38]. In addition, exercise, acting as a stress management regulator, can help control panic symptoms, which is the key for a successful, gradual withdrawal from long-term benzodiazepine use [40].

Interestingly, the beneficial effects of exercise have a cumulative, long-term impact and can be easily integrated into different clinical settings. However, while the positive effects of exercise on the afore-mentioned substance abusers have been documented individually, less is known about the possible exercise-induced effects on the concurrent use of multiple non-opioid substances and the substitute substance in patients with OUDs during medication for opioid use disorders [41].

Therefore, the aim of this study was to examine the possible effects of a two-month exercise training program on the concomitant use of multiple non-opioid substances, i.e., alcohol, cocaine, cannabis, and benzodiazepines, in patients with OUDs during their medication treatment, in order to reveal the potential of exercise as a complementary intervention to improve their treatment. The inclusion of patients with multiple substances use, opioids and non-opioids, is expected to serve as a valuable resource regarding the effects of exercise training on such addicted patients and for developing more targeted and standardized exercise interventions, rather than recreational and temporary pleasure physical activities, which are usually selected. Existing systematic reviews have included studies with a focus on alcohol, substance use, and smoking in the same analyses, as well as studies with a focus on prevention, harm reduction, and treatment [42,43]. This study examined the associations between an 8-week exercise intervention and non-opiate substance use among individuals undergoing medication for an opioid use disorder (MOUD). Although previous systematic reviews [44,45,46] have focused on opioid substitution treatment, these have been very few and are mostly limited to studies with small sample sizes [25,30]. It should be mentioned that although the number of the participants in this multiple-group design might be larger, nevertheless the sample size of this study is the largest used compared to previous studies.

## 2. Materials and Methods

### 2.1. Design, Participants, Recruitment, and Experimental Procedures

#### 2.1.1. Study Design

**Figure 1:**
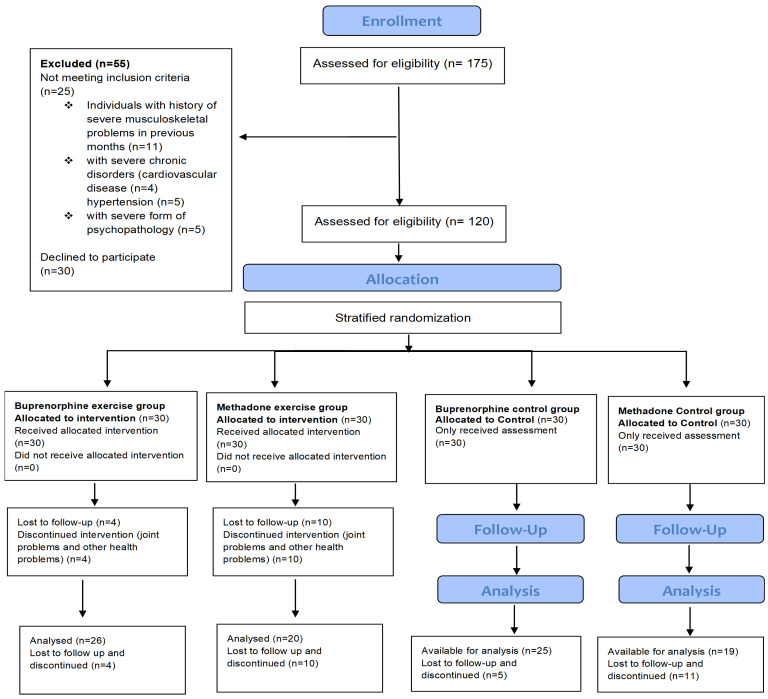
Flow chart of the recruitment, inclusion, stratified randomization, and assessment of the patients that volunteered to participate in this study.

This is a randomized controlled trial conducted in adult patients, men and women, with OUDs. The recruitment, inclusion, and stratified randomization flow diagram for the patients that volunteered to participate in this study is shown in Figure 1. One hundred and seventy-five (175) patients were recruited by the Greek Organization Against Drugs (OKANA), a treatment center of the Attica Region, between October and December 2021. The inclusion criteria were the following: (1) patients with OUDs based on the criteria of the Diagnostic and Statistical Manual of Mental Disorders (DSM-5) [47]; (2) patients coming from the OKANA stabilization phase, in which the surrogate dose had stabilized, with no pronounced withdrawal symptoms between administrations, and where the craving had been significantly reduced so as not to affect the functionality of the individual; (3) newcomers over 3 months in treatment; and (4) patients over 20 years of age. This study was implemented within the structures of OKANA, supervised by a specialized provider of health services for opioid-dependent patients, ensuring (a) an accessible location and sufficient space for exercise and the safety of the natural environment (equipment for managing an overdose or, in rare cases, aggressive behavior on the premises); (b) access to the medical records of patients in cooperation with the Heads of the Therapeutic Structures of the Organization, the facilitation for recruiting volunteers and supervising them in the exercise training program, the assessment of diagnoses, therapeutic histories, social and psychiatric status, as well as a completely updated record of the history/file of each patient participating in this research. Supervision of the exercise program is important for its efficiency, as it results in higher increases of the overall physical condition of the beneficiaries compared to unsupervised programs. Support and commitment from staff and exercise instructors have been shown to motivate adherence and participation and reduce dropout rates [30]. Moreover, supervised exercise training appears to be superior for a dependent population, as it is more effective and provides greater safety than unsupervised exercise [48].

#### 2.1.2. Exclusion Criteria and Sample Size

Participants were interviewed and their medical history was checked from their individual medical records. The exclusion criteria that made initially assessed volunteers ineligible for this study were the following: (1) severe musculoskeletal problems; (2) severe chronic diseases (e.g., hypertension, cardiovascular disease, and chronic obstructive pulmonary disease); (3) severe psychopathology (psychoses) and personality disorders (e.g., antisocial disorder); and (4) pregnancy. The exclusion criteria reduced the number of possible entries from 175 initially assessed to a final number of 120 patients, aged 21–65 years (Figure 1). A repeated measures power analysis was conducted for the sample size estimation. The power analysis was conducted for a within-subjects factor assessed over two time points. For this design, 20 participants per group achieved a power of 0.85 for the between-subjects main effect at an effect size of 0.42, a power of 0.90 for the within-subjects main effect at an effect size of 0.26, and a power of 0.90 for the interaction effect at an effect size of 0.26. The final sample size was increased to 30 per group to control for losses during follow ups. 

#### 2.1.3. Experimental Procedures

The somatometric characteristics of patients were measured, and their demographic and clinical data were collected from individual medical records. In addition, before the start of the exercise training program, three preparatory sessions of 60 min each were held to develop the patients’ trust in the research team and to increase the possibility of compliance with the program. The participants were then divided into four groups. Specifically, they were allocated to intervention and control groups by stratified randomization, utilizing computerized random numbers [49], as follows: (a) an experimental group of patients with OUDs receiving buprenorphine (n = 26), (b) an experimental group of patients with OUDs receiving methadone (n = 20), (c) a control group of patients with OUDs receiving buprenorphine (n = 25), and (d) a control group of patients with OUDs receiving methadone (n = 19). The patients of the experimental groups performed a two-month aerobic exercise training program on a treadmill, and the use of alcohol, cocaine, benzodiazepines, and cannabis was measured in days and quantity, while, at the same time, the substitute dosage was recorded to determine any possible changes. Patients in the control groups followed the usual care, and they did not exercise and received only educational information about the health benefits of exercise while gathering in a relevant environment. Finally, 90 patients (experimental groups: n = 46 and control groups: n = 44) completed this study, while 30 patients discontinued due to health or other problems.

#### 2.1.4. Ethical Approval

The study was conducted in accordance with the Declaration of Helsinki and was approved by the Ethics Committee of the Medical School of the National and Kapodistrian University of Athens (approval number: 141-27/06/2020). Also, approval for this research was provided by the Anti-Narcotics Organization, since it was conducted within its structures (approval number: 21550-31/05/2019). All participants signed a written informed consent.

### 2.2. Questionnaires, Patient history, and—Substance Use Measurements

The Treatment Outcomes Profile (TOP) questionnaire, adapted from the Australian Treatment Outcomes Profile (ATOP) scale to Greek conditions by a scientific team of OKANA executives, was utilized to monitor the clinical outcomes of the beneficiaries, in the context of therapeutic services for psychoactive substances and alcohol (D & A services) (Table 1). Specifically, this questionnaire is a tool of high validity and reliability. It monitors treatment outcomes and provides data on two main axes: (a) substance use (alcohol and other psychotropic substances) and (b) health and wellness (e.g., physical health, mental health, quality of life (QoL), work, and violence). Moreover, it is short and concise, facilitates the administration process, allows for easy and quick data collection, and it is compatible with clinical practices. The method followed for collecting information is the “Timeline Follow Back” (TLFB) [50], which aims to organize a discussion with the beneficiaries to recall substance use in detail and to record other related behaviors. It was designed specifically to evaluate changes in behavior before and after treatment. According to the TLFB method, a retrospective record of days on which it was used in each of the last four weeks is requested, starting with the most recent, and, at the same time, recording the typical amount of use (e.g., drinks, cigarettes, grams, and pills, etc.). In addition, in the first part of the questionnaire, information on the socio-demographic and clinical data of patients is given, thus having a complete picture of their profile (Table 1). The questionnaire was shared before, during, and after the completion of the exercise training program (1st, 12th, and 24th exercise session). In addition, to ensure the accuracy of the information collected, comparisons were made between patients’ responses and their clinical picture, as recorded in each patient’s individual medical record.

### 2.3. Urine Drug Monitoring

During the two-month exercise training, urine drug monitoring (UDM) records were performed, via the Drop Test Method, by OKANA’s nursing service. UDM is an important tool for checking compliance and identifying potential use and abuse in patients treated with opioids [51]. It was performed to further determine the reliability of the self-reported information collected by the TOP questionnaire, as participants’ answers were compared to the urine tests conducted during the two-month experimental period. Urine sample collection was performed weekly, in the middle of each week, to detect whether a substance had been used within the last 2–3 days before the urine sample collection. Urine drug monitoring started 31 January 2022 and ended 25 March 2022.

#### Drop Test Method Implemented in OKANA’s Structures

According to the related protocol (No: 8548-28/06/2017), toxicological tests were carried out using dry reagents and, specifically, the type with cassette morphology and dropping methodology (Drop Test). This method provides the utilization of positive and negative toxicological test results, since it is a qualitative method, but some quantitative information can be used empirically, when a faint indication appears, in the case of a very low dose. In addition, this method offers some advantages, such as easy handling, immediacy, and the short duration required for the results to be completed. Moreover, in recent years, there has been a rapid improvement in the accuracy and the effectiveness of this qualitative method. The toxicological tests carried out in the Units of OKANA checked the following psychotropic substances: opioids, benzodiazepines, cocaine, cannabis, amphetamines, alcohol, methadone, and buprenorphine. The frequency of the toxicological tests was determined in direct relation to the Treatment Phase and the Treatment Outcome Profile of the beneficiaries of the treatment programs, as defined by the Toxicological Control Protocol of OKANA.

### 2.4. Measurement of Substitute Dosage

The recording of substitute dosage was performed on the last day of each week, for a total of 8 weeks, with online information provided by the nursing service of OKANA.

### 2.5. Somatometric Measurements

Body height was measured with an accuracy of 1 cm (Seca 216, Hamburg, Germany), and body weight and fat mass were measured by a bioelectrical impedance analyzer (Tanita BC 545n, Manchester, UK).

### 2.6. Aerobic Exercise Training

The exercise training program was implemented within the therapeutic structures of OKANA, a treatment center, which beneficiaries were visiting to receive their medication therapy. Treadmills were placed in the areas where patients received their clinical treatment, and trainees exercised prior to the administration of the substitute, thus enhancing the patients’ compliance to the exercise program. Randomized controlled trials often tend to be highly supervised, which may explain why the levels of adherence are often higher compared to observational studies. In our study, a team of specialists (trainers, psychiatrists, nurses, and psychologists) were recruited for the successful completion of the program. This team of experts had a continuous presence throughout the exercise program, ensuring an adherence of the participants to it by utilizing (a) appropriate technology (continuous heart rate monitoring with electronic monitors and electrical treadmills); (b) a continuous examination of obstacles and facilitators of participants; (c) continuous briefing and updating feedback of participants on the risks and benefits of exercise; (d) in the absence of unpleasant experiences, with medical and social support, communication and constant feedback; and (e) by a continuous setting of exercise training-associated goals.

Specifically, an aerobic exercise protocol was performed on a treadmill (20 min at 70% HRmax, 3 days/week for 8 weeks). The goal was to follow an exercise protocol of moderate intensity based on the physical fitness of each participant. Initially, 70% of the maximum heart rate (HRmax) was calculated using the formula HRmax = 220 − age (yrs) × 0.7. The treadmill speed, corresponding to 70% HRmax, was calculated by measuring the HR during walking on the treadmill, starting with an initial speed of 5 km/h and increasing the speed by 1 km/h every 3 min. The heart rate was measured every 15 s during the last minute of each three-minute period. From the speed and HR recordings, the predicted treadmill speed, corresponding to 70% HRmax, of each trainee was calculated. In the first session of the exercise program, the resting HR was measured, followed by a 5 min warm-up containing breathing and stretching exercises. This was then followed by a 3 min familiarization period by walking at 80% of the target treadmill speed, and, subsequently, the participants performed the 20 min aerobic exercise protocol. The heart rate was measured every 15 s during the last minute of 5 min intervals. At the same intervals, the 20-point Borg Rating of Perceived Exertion (RPE) scale was used to assess the perceived fatigue of the patients during exercise. The end of the aerobic exercise protocol was followed by a 5 min cool-down period, again containing breathing and stretching exercises [52].

### 2.7. Statistical Analysis

All variables were first tested for normality using the Kolmogorov–Smirnov criterion [53]. Quantitative variables were expressed as means (±Standard Deviation) or as medians (interquartile range). Qualitative variables were expressed as absolute and relative frequencies. For the comparison of proportions, chi-square and Fisher’s exact tests were used. Students’ t-tests and Mann–Whitney tests were used for the comparison of continuous variables between two groups. The repeated measures analysis of variance (ANOVA) was adopted to evaluate the changes observed in alcohol consumption, tobacco use, drug use, and QoL aspects among the exercise and control groups over the experimental period. The repeated measures analysis of variance (ANOVA) was conducted after having the quantitative variables logarithmically transformed, in case of non-normal distributions. All the reported *p* values are two-tailed. Statistical significance was set at *p* < 0.05, and analyses were conducted using the SPSS statistical software (version 26.0).

## 3. Results

### 3.1. Baseline Characteristics of Participants

Of the ninety volunteers that participated in this study, 51 (56.7%) were taking buprenorphine and 39 (43.3%) methadone. Of the 51 participants under buprenorphine, 26 (51%) comprised the exercise group and 25 (49%) the control group. Of the 39 methadone participants, 20 (51.3%) comprised the exercise group and 19 (48.7%) the control group. Participants’ demographics as well as information on their substitute use are presented in Table 2. No significant differences were found between the exercise and control groups, regardless of the substitute that they were taking. The comparison between the two substitutes in the exercise groups revealed that participants using methadone were significantly older, had a greater body mass index (BMI), had a significantly lower age of first use, and had significantly more relapses during rehabilitation, while those lost at follow-ups had a higher rate of use recurrence of either opioids or non-opioid-based substances.

### 3.2. Medical History

Information on the medical history of the participants is shown in Table 2. Most of the participants exhibited simultaneously psychiatric and substance use disorders. This psychiatric coexistence was observed equally in all groups of both substitutes. More specifically, according to the medical history of patients kept in the OKANA records and based on the DSM-5 criteria, the participants showed (a) mental and behavioral disorders due to multiple drug use and the use of other psychoactive substances (F.19-N = 24); (b) mood disorders (F.30 and F.39-N = 6); (c) schizophrenia, schizotypal, and delusional disorders (F.20 and F.29-N = 3); (d) neurotic, stress-related, and somatoform disorders (F.40 and F.48-N = 22); I panic disorders (episodic paroxysmal anxiety (F.41, N = 24)); and (f) mild depressive episodes (F.32-N = 3). Overall, high rates of anxiety and depression were the most common disorders that occurred in these individuals. Of the ninety volunteers that participated in this study, 51 (56.7%) were taking buprenorphine and 39 (43.3%) methadone, and half of this population reported lifetime depression, and nearly one-third of them reported a depressed mood while receiving treatment (Table 2).

### 3.3. Changes in BZD, Cannabis, and Cocaine Use

**Figure 2:**
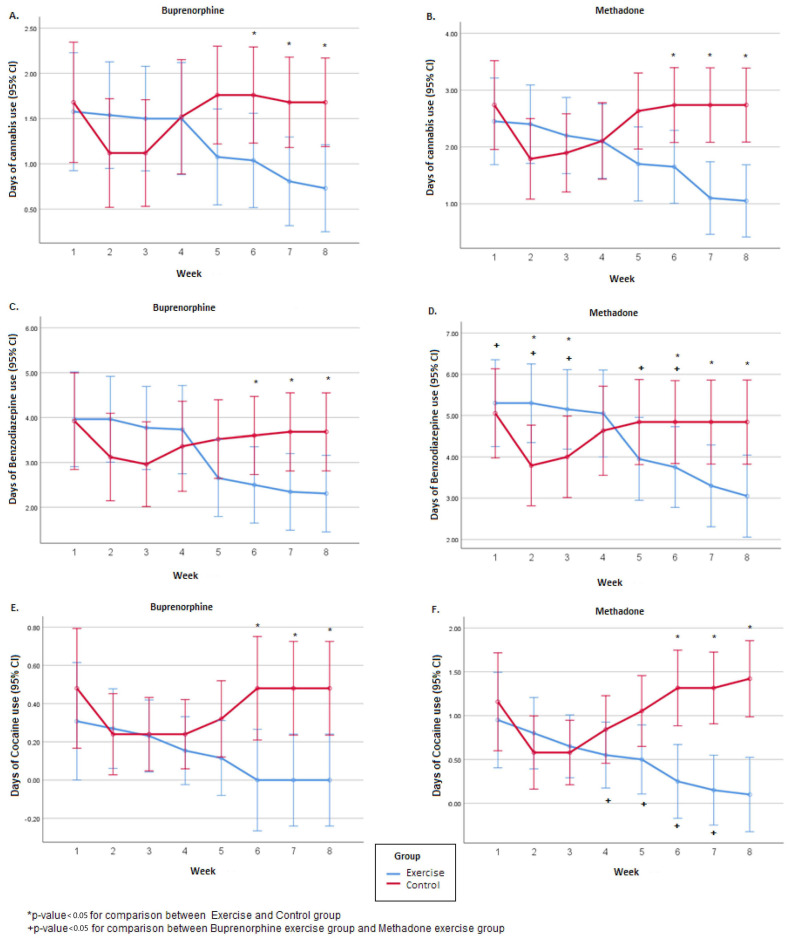
Weekly number of days of BZD, cannabis, and cocaine use during the 8-week experimental period, separated by the buprenorphine (**A**,**C**,**E**) and the methadone group (**B**,**D**,**F**).

In both substitutes, the number of days that the patients had used cannabis did not differ between the exercise and control groups during the first 5 weeks. In contrast, over the next 3 weeks, the patients in the control group used significantly more cannabis (Figure 2). In both substitutes, the total number of days during the 8 weeks of the experimental period that they used cannabis did not differ between the exercise and control groups, as did the average daily amount of cannabis during the first 4 weeks. In contrast, the average daily amount of cannabis used during the second 4 weeks, i.e., th^e^ 5th t^o^ 8th week, was significantly higher in the control groups. Overall, in both substitutes, over the 8-week course, the number of days the patients used cannabis changed significantly (F(7.343) = 5.25, *p* < 0.001 for the buprenorphine group and F(7.259) = 6.17, *p* < 0.001 for the methadone group). Also, the degree of change differed significantly between the exercise and control groups (F(7.343) = 20.74, *p* < 0.001 for the buprenorphine group and F(7.259) = 24.99, *p* < 0.001 for the methadone group). Specifically, after th^e^ 4th week, in the exercise groups, there was a decrease, and in the control groups, an increase. The comparison between the two substitutes in the exercise groups did not show any significant differences in the days of cannabis use in either the 8 weeks or overall. However, the average daily amount of cannabis used was significantly higher in people taking methadone, during both the first 4 (i.e.^,^ 1st t^o^ 4th) and the following 4 ^(^5th t^o^ 8th) weeks of the experimental period (Table 3).

In both substitutes, the total number of days during the 8 weeks of the experimental period that the patients used BZD did not differ between the exercise and control groups, nor did the average daily quantity of BZD during the first 4 weeks (Figure 2). In contrast, the average daily quantity of BZD during the second 4 weeks, i.e.^,^ 5th t^o^ 8th week, was significantly higher in the control groups. Overall, over the course of the 8-week experimental period, the number of days the patients used BZD changed significantly, regardless of the substitute (F(7.343) = 17.92, *p* < 0.001 for the buprenorphine group and F(7.259) = 16.80, *p* < 0.001 for the methadone group). Also, the degree of change was different between the exercise and the control groups (F(7.343) = 34.51, *p* < 0.001 for the buprenorphine group and F(7.259) = 40.91, *p* < 0.001 for the methadone group). Specifically, in the exercise groups, there was a decrease, and in the control groups, an increase. The comparison between the two substitutes in the exercise groups revealed that participants under methadone had used BZD significantly more over the 8 weeks. Also, the mean daily quantity was significantly higher in the methadone exercise group during both the first 4 (i.e.^,^ 1st t^o^ 4th) and the following 4 ^(^5th t^o^ 8th) weeks of the experimental period (Table 3).

In both substitutes, the number of days the patients had used COC did not differ between the exercise and control groups during the first 5 weeks. In contrast, over the next 3 weeks, the patients in the control group used significantly more COC (Figure 2). For the total days of COC use in the methadone group, the control group had significantly more days of COC use compared to the exercise group (*p* = 0.037). In the buprenorphine group, no significant difference was found between the exercise and the control group regarding the total number of days that the patients used COC (*p* > 0.05). Overall, over the course of the 8 weeks of the experimental period, the number of days the patients used COC changed significantly, regardless of the substitute (F(7.343) = 2.12, *p* = 0.033 for the buprenorphine group and F(7.259) = 3.32, *p* = 0.002 for the methadone group). Also, the degree of change was different between the exercise and the control group (F(7.343) = 7.24, *p* < 0.001 for the buprenorphine group and F(7.259) = 16.89, *p* < 0.001 for the methadone group). Sex and age effects were investigated through the repeated measurements model, and no significant differences were found (*p* > 0.05). Specifically, in the exercise groups, there was a decrease, and in the control groups, an increase. Also, no significant difference was found between the buprenorphine exercise group and the methadone exercise group regarding the total number of days that the patients used COC (*p* > 0.05) (Table 3).

### 3.4. Changes in Alcohol Use

During the 8 weeks of the experimental period, the number of days of consuming at least 5 drinks, i.e., days of hazardous/binge drinking, were similar between the exercise and control groups in both substitutes, as well as the mean daily quantity of alcohol consumption during the first 4 weeks. However, in the last 4 weeks, the exercise groups exhibited significantly lower alcohol consumption, in both the methadone and buprenorphine groups. In both substitutes, the number of days of consuming at least 5 drinks changed significantly over the 8-week period (F(7.343) = 8.41, *p* < 0.001 for the buprenorphine group and F(7.259) = 9.70, *p* < 0.001 for the methadone group), and the degree of change differed between the exercise and control groups (F(7.343) = 19.50, *p* < 0.001 for the buprenorphine group and F(7.259) = 34.27, *p* < 0.001 for the methadone group). More specifically, in both substitutes, the number of days of hazardous/binge drinking diminished in the exercise group, while in the control groups, it increased after th^e^ 3rd week. The comparison between the two substitutes in the exercise groups showed that during the first 4 weeks in the methadone group, patients consumed five or more drinks for significantly more days. At weeks 5 to 8, there were no significant differences in the number of days of hazardous/binge drinking between the two substitutes. The total number of days that the patients consumed five or more drinks was significantly higher in the methadone exercise group as was also the average daily quantity of alcohol during weeks 1–4 and 5–8 (Table 4).

### 3.5. Changes in Dosage of Medication for Opioid Use Disorders

The dosage of medication for opioid use disorders differed significantly between the buprenorphine exercise group and the buprenorphine control group during the 6th and 7th week of the experimental period (*p* = 0.007 and *p* = 0.022, respectively) (Table 5). Specifically, the substitute dosage was reduced during the 6th and 7th week only in BEX. Between the methadone exercise group and the methadone control group, no significant differences were found throughout the 8-week experimental period. Although direct equivalence between buprenorphine and methadone is difficult to estimate and there is not a linear relationship between them, previous studies have shown that 8 mg of sublingual buprenorphine are equivalent to 60 mg of oral methadone. Others suggest that 12 to 16 mg of buprenorphine is approximately as effective as 50 to 80 mg of methadone [54,55,56].

### 3.6. Changes in Health and Welfare

#### 3.6.1. Days of Work and Education

Overall, over the 8 weeks of the experimental period of this study, the number of working days increased regardless of the substitute (F(7.343) = 13.87, *p* < 0.001 for the buprenorphine group and F(7259) = 10.57, *p* < 0.001 for the methadone group). However, the degree of change differed between the exercise and the control groups in both substitutes (F(7.343) = 2.79, *p* = 0.008 for the buprenorphine group and F(7.259) = 2.53, *p* = 0.015 for the methadone group), with the increase in the exercise groups being greater. In addition, over the period of 8 weeks, the total number of education days also increased regardless of the substitute (F(7.343) = 15.14, *p* < 0.001 for the buprenorphine group and F(7.259) = 4.86, *p* < 0.001 for the methadone group). However, the degree of change differed between the exercise and the control group only in the methadone group (F(7.259) = 2.53, *p* = 0.015). Specifically, in the exercise group, there was an increase, while in the control group, no change was observed (Table 6).

#### 3.6.2. Sports/Voluntary Days

In the buprenorphine group, over the 8 weeks, the total number of sports/voluntary days increased (F(7.343) = 23.98, *p* < 0.001), and the degree of change was different between the two groups (F(7.343) = 89.29, *p* < 0.001). Specifically, in the exercise group, there was an overall increase, while in the control group, there was an initial increase only during the 2nd and 3rd week and then the number of sports/voluntary days decreased close to the baseline levels. In the methadone group, over the 8 weeks of the experimental period, the number of sports/voluntary days increased significantly (F(7.259) = 20.81, *p* < 0.001), while the degree of change was significantly different between the two groups (F(7.259) = 54.41, *p* < 0.001). Specifically, in the exercise group, there was an overall increase, while in the control group, there was an initial increase during the 2nd and 3rd week and then the number of sports/voluntary days gradually decreased to the baseline levels. Moreover, the comparison between the two substitutes in the exercise groups revealed that patients on methadone had significantly fewer sports/voluntary days over the 8-week period (Table 7).

#### 3.6.3. Homelessness/Violence/Arrest Days

In the buprenorphine group, throughout the 8-week experimental period, the number of homelessness/violence/arrest days was significantly larger in the control group compared to the exercise group. Moreover, the number of days of homelessness/violence/arrest changed significantly over time (F(1.49) = 23.29, *p* < 0.001), while the degree of change was different between the two groups (F(1.49) = 23.29, *p* < 0.001). Specifically, in the exercise group, there was a decrease, while in the control group, no significant changes were observed. In the methadone group, the number of homelessness/violence/arrest days was significantly higher in the control group in the second half of the program (i.e., week 5–8), compared to the exercise group, while in the first half, there was no significant difference. Overall, over the course of 8 weeks, the number of homelessness/violence/arrest days significantly decreased (F(1,37) = 54.61, *p* < 0.001). The degree of change differed significantly between the two groups (F(1,37) = 37.20, *p* < 0.001). Specifically, in the exercise group, there was a decrease, while in the control group, there was no significant change. Moreover, the comparison between the two substitutes in the exercise groups showed that the number of homelessness/violence/arrest days was significantly larger in methadone patients compared to buprenorphine ones in both the first 4 (i.e., 1st to 4th) weeks and the following 4 (5th to 8th) weeks of the experimental period (Table 7).

**Table 7:** Participants’ health and welfare during the 8-week experimental period.

	Buprenorphine				*p* ^1^	Methadone				*p* ^1^	*p* ^2^
	Exercise Group		Control Group			Exercise Group		Control Group			
	Mean (SD)	Median (IQR)	Mean (SD)	Median (IQR)		Mean (SD)	Median (IQR)	Mean (SD)	Median (IQR)		
Days with sports/volunteering											
1st week	1.58 (1.1)	1.5 (1–3)	1.4 (0.96)	1 (1–2)	0.556	0.7 (0.73)	1 (0–1)	0.63 (0.68)	1 (0–1)	0.793	0.007
2nd week	1.65 (1.13)	2 (1–3)	2.84 (1.14)	3 (2–3)	0.001	0.85 (0.88)	1 (0–1)	1.79 (1.03)	2 (1–2)	0.004	0.016
3rd week	1.65 (1.13)	2 (1–3)	2.32 (1.25)	3 (1–3)	0.050	0.85 (0.88)	1 (0–1)	1.42 (0.96)	1 (1–2)	0.057	0.016
4th week	1.77 (1.24)	2 (1–3)	1.68 (1.03)	2 (1–2)	0.838	0.9 (0.91)	1 (0–1.5)	0.63 (0.83)	0 (0–1)	0.331	0.016
5th week	2.04 (1.31)	2 (1–3)	1.4 (0.96)	1 (1–2)	0.066	1.05 (0.89)	1 (0–2)	0.53 (0.7)	0 (0–1)	0.053	0.009
6th week	2.5 (1.39)	3 (2–4)	1.48 (0.96)	1 (1–2)	0.005	1.15 (0.93)	1 (0–2)	0.53 (0.7)	0 (0–1)	0.031	0.001
7th week	2.92 (1.38)	3 (2–4)	1.32 (0.85)	1 (1–2)	<0.001	1.6 (0.88)	2 (1–2)	0.53 (0.7)	0 (0–1)	<0.001	0.001
8th week	3.15 (1.35)	3 (2–4)	1.32 (0.85)	1 (1–2)	<0.001	2 (0.86)	2 (1–2.5)	0.53 (0.7)	0 (0–1)	<0.001	0.003
1th–8th week	17.27 (9.66)	19 (11–26)	13.76 (7.46)	12 (9–19)	0.112	9.1 (6.56)	9.5 (2–12.5)	6.58 (5.78)	4 (3–10)	0.324	0.002
Homeless/Violence/ Arrest											
1st–4th week	1.96 (0.53)	2 (2–2)	2.08 (0.64)	2 (2–2)	0.462	4.2 (1.36)	4 (3.5–5)	4.21 (1.13)	4 (4–5)	0.988	<0.001
5th–8th week	1.35 (0.49)	1 (1–2)	2.08 (0.64)	2 (2–2)	<0.001	3.1 (1.12)	3 (2–4)	4.11 (1.1)	4 (4–5)	0.010	<0.001
1th–8th week	3.31 (0.79)	3 (3–4)	4.16 (1.28)	4 (4–4)	0.005	7.3 (2.41)	7 (6–9)	8.32 (2.21)	8 (8–10)	0.202	<0.001
Physical and mental health/QoL											
1st week	7 (1.13)	7 (6–8)	6.92 (0.95)	7 (6–7)	0.835	4.9 (1.12)	5 (4–6)	4.74 (1.37)	5 (3–6)	0.812	<0.001
4th week	13.12 (1.88)	13.5 (12–14)	11.2 (2.04)	12 (9–12)	0.001	11.1 (1.48)	11 (10–12)	7.47 (1.74)	7 (6–10)	<0.001	<0.001
8th week	19.12 (2.08)	19 (18–21)	12.6 (2.53)	13 (11–14)	<0.001	17.5 (1.73)	17.5 (16.5–18)	9.68 (2.14)	9 (8–12)	<0.001	0.011
1th–8th week	39.23 (4.47)	41 (35–43)	30.72 (5.06)	32 (26–34)	<0.001	33.5 (3.65)	33 (32–34)	21.89 (4.81)	21 (18–28)	<0.001	<0.001

*p*^1^—value for comparison between exercise and control group (Mann–Whitney test). *p*^2^—value for comparison between buprenorphine exercise group and methadone exercise group (Mann–Whitney test).

#### 3.6.4. Quality of Life

Regardless of the substitute used, the QoL was similar at week 1 in all, the exercise and the control, groups. However, at weeks 4 and 8, there were significant differences, with the patients in the exercise groups exhibiting a better QoL, while, overall, the QoL in the exercise groups was also significantly better. Specifically, over the 8-week experimental period, the QoL increased significantly in the exercise groups compared to the control groups (F(2.98) = 745.30, *p* < 0.001 for the buprenorphine group and F(2.74) = 790.77, *p* < 0.001 for the methadone group), while the degree of increase was significantly different between the two groups (F(2.98) = 102.5, *p* < 0.001 for the buprenorphine group and F(2.74) = 150.83, *p* < 0.001 for the methadone group). Moreover, the comparison between the two substitutes in the exercise groups showed that the QoL of those taking buprenorphine was significantly better compared to those taking methadone, throughout the experimental period (Table 7).

## 4. Discussion

This is the first study investigating the effectiveness of exercise training intervention on reducing concurrent non-opioid substance use in patients with OUDs under substitution treatment. Our main findings were that after the two-month exercise training in opioid users during maintenance treatment, the parallel non-opiate drug (alcohol, cocaine, cannabis, and benzodiazepines) use decreased significantly in the exercise groups of both methadone and buprenorphine substitution compared to the controls. The randomized controlled trial design of this study demonstrated that the reductions in substance use observed in the experimental (exercise) groups were not a result of the substitution medications. Also, the dosage of medication used for OUDs differed significantly between the buprenorphine exercise group and the buprenorphine control group during the 6th and 7th week of the experimental period (Table 5). Specifically, the substitute dosage was reduced during the 6th and 7th week in both BEX and MEX, while no significant differences were observed in either of the control groups throughout the 8-week experimental period. There are studies showing that 100 mg of methadone or more and a buprenorphine dose of 16 mg and above are better for reducing recurrence of use and for preventing death [57]. This is particularly important as fentanyl use spreads and higher doses of MOUDs are required to reduce cravings, recurrence of use, and overdose deaths [58]. However, in this regard, and according to our data, there was no difference in the abandonment and dose of MOUDs, which were decreased because of exercise. This indicates that exercise can help patients achieve recovery when combined with evidence-based pharmacological treatments such as buprenorphine or methadone [48]. Although opiate agonists are an effective intervention for opiate dependence, there is room for improvement, since many patients using opiate agonists continue to use also other substances, have comorbid psychiatric problems, and experience a poor quality of life [59]. Engaging in alternative activities, such as exercise, combined with medication therapy for OUDs appears to be associated with an improved treatment [34].

In the present study, the participants were newcomers to OKANA’s programs (up to 3 months), the sample was gender balanced, and the comparison between the two substitutes used in the exercise groups revealed that patients with methadone use exhibited more relapses during recovery and a reduced response to exercise compared to the buprenorphine group. The older age and higher body mass index (BMI) of the methadone group might account for the exercise differences compared to the buprenorphine group. Despite the efficacy of methadone for opioid dependence, an adverse effect of methadone use may be weight gain [60]. Overall, the potential weight-increasing effects of methadone are further associated with negative psychosocial impacts, such as a low self-esteem and societal stigmatization and an impaired metabolic function, eventually resulting in an increased BMI of MOUD patients [60,61,62,63,64,65]. These effects may contribute to the high rates of nonadherence to a methadone maintenance treatment (MMT) [66,67]. Moreover, older adults make up an increasing proportion of those receiving MMTs for OUDs [68], and many health-related problems, prevalent in elderly populations that receive MMTs, are associated with several adverse effects, which might account for the exercise differences in the buprenorphine group [69]. A better understanding of the benefits and barriers for exercise among the vulnerable subpopulation of substance abusers could lead to the development of optimum exercise training protocols with important clinical implications for MMT patients.

Moreover, in our study, patients that used buprenorphine rated their physical function significantly higher than the ratings of the patients that used methadone, also reporting a higher psychological function and an overall improvement in QoL. These findings are in line with those of recent studies that indicate the advantages of buprenorphine vs. methadone treatment in terms of physical and psychological functions as well as QoL improvements [65,66]. Patients and clinicians have begun to recognize the advantages and disadvantages of buprenorphine over methadone, but the factors influencing choices between these two drugs remain unclear. It is possible that patients’ preferences and past experiences influence treatment decisions. However, a related analysis revealed seven areas of interest in the decision making for opioid agonist treatment: (1) the awareness of treatment options, (2) expectations and goals for treatment durations and abstinence, (3) previous experience with buprenorphine or methadone, (4) the need for accountability and structured support, (5) the preference to avoid methadone clinics or associated stigma, (6) the fear of continued addiction and perceived difficulty with withdrawal, and (7) pain control [70]

In patients with OUDs, psychiatric and substance use disorders usually coexist and may be interconnected, as a substance use disorder may worsen psychiatric symptoms, while psychiatric disorders may trigger substance use [70]. This association may reflect a causal relationship between these disorders or an underlying vulnerability of the individual to both disorders [71]. Unfortunately, psychiatric comorbidity in patients already suffering from substance use disorders is often associated with a poorer commitment to treatment, usually leading to a relapse of substance use [72] and an increased recovery time [73]. In the present study, most patients were characterized by a high rate of comorbidities, i.e., the simultaneous occurrence of two or more diseases, such as alcohol dependence and depression.

Regular aerobic exercise could modulate a variety of psychological symptoms in addicts, such as anxiety, depression, paranoia, hostility, or compulsion, and thus could effectively improve the physical condition, emotional state, and mental health of addicts, as well as reduce their drug addiction [74]. Although patients of low severity seem to benefit more from exercise interventions than those of higher severity, the exercise prescription should be personalized and its outcomes should be evaluated so as to achieve the objectives [75]. It has been reported that people with opioid use disorders are interested in exercising as part of their treatment to reduce their desire for substance use and improve their overall physical and mental health [76]. Moreover, those patients prefer to start an exercise program early in their recovery (i.e., in the first 3 months) [77,78,79].

Interestingly, there are studies that support that citizenship/community involvement, engaging in meaningful activities, and housing/safety are linked to the recovery of these patients [80,81,82]. The satisfaction of the above needs can contribute decisively to a further destigmatization of patients with OUDs so that they are healthy and functional in their family, professional, and social space, while at the same time, the community will enjoy their participation and contribution. The management of structured exercise interventions in patients with OUDs can be complicated due to homelessness, social instability, and unemployment. However, as exercise becomes regular, it acts as a mediator of shaping a healthy everyday life and is the link for the participation of those individuals in meaningful activities, since it promotes increased social inclusion while preventing loneliness and isolation. Indeed, after the two-month exercise intervention, there were improvements in both experimental groups regarding physical and mental health and the adoption of a quality lifestyle, without violence and crime, while exercise training also contributed to the satisfaction of three basic needs of the addicts, i.e., work, education, and housing, which are key factors for a truly successful treatment. These findings are in accordance with those of a recent study [83], showing a significant positive relationship between work status, educational attainment, and the simultaneous use of multiple drugs, concluding that effective approaches, including campaigns to promote healthy behaviors, can act as facilitators in this positive relationship, and, hence, they should be integrated into programming aimed at reducing rates of opioid use and the concomitant use of multiple drugs among patients on opioid replacement therapy [83,84]. In addition, lifestyle-focused interventions, such as exercise, have the potential to increase the search for help by reducing the perceived stigma experienced by this category of patients [41]. Indeed, it has been shown that exercise could positively affect the emotional state of addicts, weakening feelings of shame and inferiority, properly reshaping ideals and beliefs, improving the sense of well-being, and eventually helping them to improve their mental health and QoL [85].

In this study, those individuals lost at follow-ups exhibited a higher rate of recurrence of use in either opioids or non-opioid-based substances. Indeed, most individuals do not adhere to or sustain a regular physical activity schedule, and about 50% drop out or do not adhere to their exercise regimen [86]. The most common reasons reported for not adhering or dropping out of exercise programs are health-related issues and a relapse to substance use [42]. Drop outs in exercise programs appear to be associated with drop outs in opioid substitution therapy [48], and, thus, an emphasis should be placed on the compliance with exercise programs.

Numerous clinical studies have utilized different types of exercise/physical activities to treat people with substance use disorders, including yoga, weightlifting, swimming, team sports, and outdoor recreational activities [87,88,89,90]. However, related research highlights the need for more standardized exercise interventions, characterized by a stronger methodology [30] compared to recreational and temporary pleasures, usually preferred in this clinical population. Aerobic exercise is a promising, non-pharmacological treatment currently being considered as a therapeutic strategy in patients with a concomitant use of alcohol, benzodiazepines, cannabis, and cocaine during opioid replacement therapy. Moreover, its effectiveness as a treatment has been shown to depend on its intensity and quantity, as well as on the stage of treatment and the type of substance abused [91], and it has been speculated that the amount or intensity of exercise may have a “more targeted effect” in terms of reducing drug cravings in people with substance use disorders (SUDs) [92]. More specifically, medium amounts of physical exercise are effective for people with substance use disorders (SUDs) to improve their internal inhibition and thus reduce and alleviate their cravings for drugs [92]. Additionally, short periods of exercise can be an effective strategy to reduce cravings, prevent drug relapses, and promote retention in treatment [41]. The treatment stage at which exercise begins has also been shown to be important for the effectiveness of exercise [93].

In the two-month aerobic exercise protocol used in this study, such specific characteristics were utilized to increase the possibility of achieving the above-mentioned beneficial outcomes, i.e., regular aerobic exercise (3 days/week) of moderate intensity (70% HRmax) for an adequate duration (8 weeks), while the total amount of training, the break time, the estimated maximum oxygen uptake (VO_2_ max) and the overall physical condition of each patient, as well as his/her stage of treatment and the type of addictive substance used, were taken into account. Moreover, the exercise training protocol was easily accessible, was integrated in the clinical settings (i.e., in places where the patients were receiving their clinical treatment), and was applied in parallel with the patients’ treatment to ensure its implementation concurrently with the substitution-substance use. In addition, the exercise training-induced decreases in the concurrent use of non-opiate drugs in both the methadone and buprenorphine substitution groups were verified with the urine tests of the participants’ samples that were collected during the two-month exercise training program. Specifically, in the control groups, during the first two weeks, a significant decrease in alcohol, benzodiazepine, cannabis, and cocaine use was observed possibly due to the educational awareness about the health benefits of exercise (behavioral approach). This decrease was accompanied by excessive enthusiasm and overconfidence of the patients for a better therapeutic outcome. However, after the third week, the participants’ enthusiasm and confidence was lost and the consumption of alcohol, benzodiazepines, cannabis, and cocaine returned to the baseline levels, while in some cases, it increased at the end of 8 weeks compared to the initial values. Although the positive effects of behavioral approaches in the treatment of substance abuse disorders, as well as the high rates of relapse, might be expected [94], this is the first study showing that, despite the initial positive effects of behavioral therapy, relapse occurs later after its start.

In contrast, in the exercise groups, there was a gradual decrease in the consumption of alcohol, benzodiazepines, cannabis, and cocaine throughout the 8 weeks of the experimental period, with the decrease being greater during the last four weeks of the intervention. These results suggest that the beneficial effects of exercise training occur gradually and support the view of a sequential (temporal) consolidation of exercise functions in favor of gradual disengagement from addiction [95]. Specifically, the sequence in which the mechanisms induced by exercise are unified follows the model of Internal Activation—Self-Regulation—Commitment, with the ultimate result of modifying the molecular and behavioral aspects of addiction [95]. Moreover, the impact of exercise on the improvement of opioid-dependent patients is more obvious when aerobic regimens of moderate intensity are applied [95], while these forms of exercise may also promote and strengthen behavioral therapeutic approaches and lead to reduced substance-consumption outcomes [94].

Although there is relatively sufficient evidence regarding the effects of exercise on each addictive substance individually, data on the possible effects of exercise on the concomitant use of multiple substances in addition to the substitute substance are limited. Users of one drug usually concurrently use other types of drugs. Nevertheless, it might be useful to discuss separately the exercise training-induced changes in their usage, as the effectiveness of exercise appears to be related to the type of addictive substance and potentially drug-specific addictive mechanisms.

### 4.1. Alcohol

Weekly alcohol consumption (days) decreased in both exercise groups after the two-month exercise intervention in this study, with the decrease being greater in the buprenorphine group compared to the methadone group, while there were no changes in any of the control groups, compared to the baseline levels. Similarly, the daily amount of alcohol intake (drinks) decreased after the intervention in both exercise groups (BEX and MEX), while it did not differ in the control groups (BCON and MCON). This beneficial effect of exercise training is very important and may indicate other exercise-induced preventive benefits, since the effects of some psychoactive substances may escalate the risk of using other substances [96,97,98,99,100,101].

A growing body of research has focused on the use of aerobic exercise as a non-pharmacological adjunct therapy in patients with alcohol use disorders. Specifically, an association between the reduction in alcohol consumption and the adherence to exercise therapy has been reported after 12 weeks of aerobic exercise intervention [102]. In addition, men who drank too much alcohol and participated in an 8-week aerobic exercise training program exhibited a decrease in alcohol intake and an improvement in their general health [103]. Interestingly, aerobic exercise has even been shown to reduce alcohol-induced structural damage to the white matter of the upper longitudinal sheath and outer capsule [104]. Overall, the therapeutic role of physical exercise and particularly aerobic exercise regimens can exert a double action on alcoholism, since it can mitigate the negative effects of alcoholism on health, while at the same time acting on the central neurotransmitter systems involved in the mechanisms of addiction [105].

### 4.2. Cocaine

Moreover, in our study, weekly cocaine consumption (days) decreased in both exercise groups after the two-month exercise training intervention, with the decrease being higher after week 5, while there were no changes in both control groups compared to the baseline levels. These findings are in accordance with those of a previous study, which showed that aerobic exercise for four consecutive weeks tended to reduce cocaine consumption during the 24 h after each exercise session, although this reduction was not significant [106]. Cocaine addiction, in combination with alcohol, has been reported to increase cravings of these addicts [107,108,109]. Thus, the decreased weekly cocaine consumption observed after the submaximal aerobic exercise protocol (70% HRmax) utilized in the present study might be attributed to an exercise-induced internal inhibition of cravings for cocaine [92]. Moreover, the positive effects of aerobic exercise may be neuroprotective due to the potential of aerobic training to restore synaptic deficits caused by cocaine [37].

In addition, it has been reported that cocaine is a stimulant drug exerting physical effects that include an increased heart rate and blood pressure, which, in the long term, could lead to serious adverse cardiovascular consequences, which may be exacerbated by strenuous physical activity [110]. Moderate-intensity aerobic exercise, as the protocol used in this study, has not been associated with side effects and is an effective strategy against many physical health problems, including heart disease [106]. More specifically, a decrease in the resting heart rate as well as an improved overall fitness have been documented in cocaine users after completing a 4-week moderate aerobic exercise training intervention [106].

Also, it should be mentioned that people who are addicted to cocaine or its combinations with alcohol, opioids, and benzodiazepines [111] exhibit altered nervous, behavioral, and physiological reactions to stress. Interestingly, recent research has shown that regular aerobic exercise can alter the brain’s dopamine mesolimbic pathway, which is linked to the rewarding and enhancing properties of drugs like cocaine [112]. Preclinical studies have also reported the effectiveness of aerobic exercise as a neuroprotective treatment and for reducing cocaine self-administration and drug-seeking behaviors [108,113,114,115,116].

### 4.3. Cannabis

After the two-month exercise training intervention, the weekly cannabis consumption in days and quantity decreased in both exercise groups, with the decrease being greater after week 5, while no significant changes were found in both control groups compared to the baseline levels. These findings add new information regarding the beneficial effects of aerobic exercise in patients with cannabis concomitant use during medication for opioid use disorders. A previous pilot study examined the feasibility of a moderate-intensity 12-week aerobic exercise program in an outpatient environment with 31% cannabis use, showing a significant increase in the percentage of abstinence days by the end of the trial [117]. Another study found that 10 days of moderate-intensity aerobic exercise for 30 min per exercise session led to significantly reduced levels of cannabis consumption and daily cravings in people not seeking treatment [38]. Since synthetic cannabinoids are new and often evolving drugs, their long-term effects on the brain remain to be established. There are only some preliminary reports indicating that their psychoactive power and potential abuse is much greater than that of natural cannabis [38]. In addition, there are findings suggesting that cannabis and cocaine use result in decreased respiratory muscle strength and the ability to exercise in these patients compared to healthy individuals [118]. Aerobic exercise regimens, such as the one used in our study, can increase lung vital capacity and contribute to the improvement of cardiopulmonary function and fitness in addicts [119].

### 4.4. Benzodiazepines

People suffering from sleep problems, mood swings, anxiety, physical and/or mental health problems, reduced euphoria, panic attacks, or drug withdrawal symptoms may crave the effects of combining benzodiazepines and opioids [120,121,122]. In addition, multiple benzodiazepines use in parallel with methadone and buprenorphine is a very dangerous drug combination that can have a fatal outcome [29,123]. Successful withdrawal strategies should combine gradual dose reduction and psychological support [124].

Nevertheless, studies have shown that people with satisfactory fitness levels are up to 15% less likely to seek out powerful drugs such as benzodiazepines, while regular aerobic exercise of moderate intensity is the best strategy to achieve this goal [125]. Specifically, it has been documented that aerobic exercise affects adrenaline levels and causes the release of endorphins that are effective in reducing anxiety, depression, and cravings in these addicts [74,126,127]. The findings of our study are compatible with such exercise-induced effects, since after the two-month exercise training, weekly benzodiazepine consumption decreased in days and quantity in both exercise groups, with the decrease being higher after week 4, while there were no significant changes in benzodiazepine consumption in the control groups compared to their baseline levels. Overall, the results of this study revealed the beneficial effects of exercise on the concurrent use of multiple non-opiate substances, corroborating the findings of previous studies regarding the positive effect of exercise on each addictive substance individually. Despite the strengths of this study, certain limitations should be considered when interpreting its findings. Specifically, all participants were middle-aged adults, with an average age of 41.9 years for the methadone groups and 46.5 years for the buprenorphine groups. Future research should include individuals of a wider age range to reveal potential age-related variations regarding the effects of exercise training on patients with OUDs during their medication therapy. In addition, all participants were characterized by opioid use disorders and followed substitute treatments; thus, the findings of this study cannot be generalized to other categories of addicts or patients with OUDs that received other types of treatment. Also, in this study, we did not examine synthetic cannabinoid use because of its newness, and, thus, there is a need for this to be included in future studies. Moreover, no drug-related factors evaluated in this study, such as work, education, homelessness, violence, and arrests, were self-reported by the participants, and, thus, they must be assessed with caution. In addition, the assessment of a combination of work/education/and volunteer days, to assess positive alternatives of patients to spend their time as well as the potential interactions between these alternatives, was not performed in this study. Finally, potential differences in physical activity levels between the participants, as a confounder to our intervention, was not directly measured.

## 5. Conclusions

Physical exercise is a useful non-pharmaceutical intervention for the improvement of numerous variables related to the recovery of opioid-dependent patients. The findings of this study confirm the value of aerobic exercise of moderate intensity for being integrated in the clinical practice for the treatment of concurrent non-opiate substance use in opioid-dependent patients under substitution treatment. It is a low-cost, feasible, and non-stigmatizing intervention that is not associated with any side effects and that can help abusers of multiple addictive substances to improve their physical and mental health.

## Figures and Tables

**Table 1:** Questionnaire used for monitoring clinical outcomes regarding psychoactive substance and alcohol use before, during, and after the completion of the experimental period in the experimental and control groups.

Treatment Outcome Profile HTOP	Data Type
**History of Drug Use:**	
Age at onset of drug intake (year)	Quantitative
2. Duration of drug intake (years)	Quantitative
3. Type of Drug Use: (a) Alcohol (b) cannabis (c) cocaine (d) benzodiazepine (e) amphetamines (f) heroin and other opiates (g) tobacco products (h) other drugs	Multi-selection
4. Frequency of drug use (days/week)	Quantitative
5. Mean Daily Quantity of drug use: typical amount of use (e.g., drinks, cigarettes, grams, pills, etc).	Quantitative
6. Beginning of Treatment	Quantitative
7. Substitute Dosage (mg): At the last day of each week	Quantitative
**Health and Welfare**	
Client’s Mental Health Status	Single-selection
2. Client’s Physical Health Status	Single-selection
3. Client’s General Quality of Life	Single-selection
4. Days of paid labor, education or vocational training or sport/volunteering	Quantitative
5. Have been (a) homeless (b) in danger (c) arrested	Single-selection

**Table 2:** Baseline characteristics of participants.

	Buprenorphine		*p* ^1^	Methadone		*p* ^1^	*p* ^2^
	Exercise Group	Control Group		Exercise Group	Control Group		
	Ν = 26; 28.9%	Ν = 25; 27.8%		Ν = 20; 22.2%	Ν = 19; 21.1%		
	Ν (%)	Ν (%)		Ν (%)	Ν (%)		
Age (years), mean (SD)	41.9 (6.1)	41.9 (5.6)	0.983 ^+^	46.7 (6.6)	46.1 (7.5)	0.794 ^+^	0.014 ^+^
Gender							
Men	15 (57.7)	11 (44.0)	0.328 ^++^	12 (60.0)	11 (57.9)	0.894 ^++^	0.875 ^++^
Women	11 (42.3)	14 (56.0)		8 (40.0)	8 (42.1)		
BMI (kg/m^2^), mean (SD)	23 (2.4)	22.9 (2.3)	0.911 ^+^	25 (3.4)	25.3 (1.8)	0.742 ^+^	0.019^+^
BMI (kg/m^2^)							
Normal	22 (84.6)	16 (64.0)	0.091 ^++^	9 (45.0)	8 (42.1)	0.855 ^++^	0.004 ^++^
Overweight/Obese	4 (15.4)	9 (36.0)		11 (55.0)	11 (57.9)		
Age at first use, mean (SD)	21 (2.9)	22.2 (3.3)	0.175 ^+^	17.5 (2.5)	18.2 (3.2)	0.449 ^+^	<0.001 ^+^
Diagnosis							
F.19	5 (19.2)	2 (8.0)	-	10 (50.0)	7 (36.8)	-	-
F.19–F.30	0 (0.0)	0 (0.0)		1 (5.0)	0 (0.0)		
F.19–F.32	0 (0.0)	0 (0.0)		1 (5.0)	2 (10.5)		
F.19–F.40	1 (3.8)	0 (0.0)		3 (15.0)	2 (10.5)		
F.19–F.41.1	2 (7.7)	2 (8.0)		0 (0.0)	0 (0.0)		
F.20–7.29	0 (0.0)	0 (0.0)		1 (5.0)	2 (10.5)		
F.30–F.39	0 (0.0)	0 (0.0)		1 (5.0)	2 (10.5)		
F.40–F.48	5 (19.2)	10 (40.0)		3 (15.0)	4 (21.1)		
f.41	5 (19.2)	2 (8.0)		0 (0.0)	0 (0.0)		
F.41	7 (26.9)	7 (28.0)		0 (0.0)	0 (0.0)		
F.41–F.39	1 (3.8)	2 (8.0)		0 (0.0)	0 (0.0)		
HIV	3 (11.5)	0 (0.0)	0.080 ^++^	6 (30.0)	2 (10.5)	0.235 ^‡‡^	0.149 ^‡‡^
Hepatitis	3 (11.5)	4 (16.0)	0.703 ^‡‡^	4 (20.0)	0 (0.0)	0.106 ^‡‡^	0.682 ^‡‡^
Number of relapses during rehabilitation. Mean (SD)	1 (1–2)	1 (1–2)	0.802 ^‡^	4 (3–4)	4 (3–4)	0.767 ^‡^	<0.001 ^‡^

^+^ Student’s t-test. ^‡^ Mann–Whitney test ^++^ Pearson’s chi-square test ^‡‡^ Fisher’s exact test. *p*^1^—value for comparison between exercise and control group. *p*^2^—value for comparison between buprenorphine exercise group and methadone exercise group.

**Table 3:** Cannabis, BZD, and cocaine use by participants during the 8-week experimental period.

	Buprenorphine				*p* ^1^	Methadone				*p* ^1^	*p* ^2^
	Exercise Group		Control Group			Exercise Group		Control Group			
	Mean (SD)	Median (IQR)	Mean (SD)	Median (IQR)		Mean (SD)	Median (IQR)	Mean (SD)	Median (IQR)		
Cannabis use											
Total number of days	9.77 (11.08)	0 (0–20)	12.32 (10.68)	14 (0–21)	0.384	14.65 (11.1)	18.5 (0–24)	19.37 (11.09)	21 (13–27)	0.245	0.110
Mean daily quantity (1st–4th week)	1.31 (1.49)	0 (0–3)	1.68 (1.52)	2 (0–2)	0.560	2.3 (1.69)	3 (0–4)	2.63 (1.38)	3 (2–4)	0.683	0.033
Mean daily quantity (5th–8th week)	0.65 (0.8)	0 (0–1)	1.6 (1.35)	2 (0–2)	0.010	1.5 (1.28)	2 (0–3)	2.53 (1.31)	3 (2–3)	0.016	0.022
Benzodiazepine use											
Total number of days	25.23 (16.89)	32 (0–40)	27.84 (19.38)	37 (0–42)	0.336	34.85 (15.46)	39.5 (26–44.5)	36.84 (18.53)	45 (24–50)	0.311	0.031
Mean daily quantity (1st–4th week)	1.58 (1.03)	2 (0–2)	1.52 (1)	2 (0–2)	0.800	2.25 (0.91)	2 (2–3)	2.11 (1.05)	2 (2–3)	0.735	0.012
Mean daily quantity (5th–8th week)	0.77 (0.51)	1 (0–1)	1.44 (1)	2 (0–2)	0.005	1.45 (0.69)	2 (1–2)	2 (1.11)	2 (1–3)	0.035	0.001
Cocaine											
Total number of days	1.08 (2.48)	0 (0–0)	2.96 (5.41)	0 (0–0)	0.424	3.95 (5.77)	0 (0–9.5)	8.26 (7.38)	12 (0–14)	**0.037**	0.105

Note. Daily quantity of cocaine was not recorded. *p*^1^-value for comparison between exercise and control group (Mann–Whitney test). *p*^2^-value for comparison between buprenorphine exercise group and methadone exercise group (Mann–Whitney test).

**Table 4:** Alcohol consumption by participants during the 8-week experimental period.

	Buprenorphine				*p* ^1^	Methadone				*p* ^1^	*p* ^2^
	Exercise Group		Control Group			Exercise Group		Control Group			
	Mean (SD)	Median (IQR)	Mean (SD)	Median (IQR)		Mean (SD)	Median (IQR)	Mean (SD)	Median (IQR)		
Days consuming at least 5 drinks											
1st week	2.62 (1.63)	3 (2–4)	2.8 (1.58)	3 (2–4)	0.869	4.05 (1.67)	4 (3–5)	4.21 (1.44)	4 (3–5)	0.772	0.007
2nd week	2.54 (1.58)	3 (2–4)	2.16 (1.21)	2 (2–3)	0.168	4 (1.59)	4 (3–5)	3 (1.2)	3 (2–4)	0.023	0.003
3rd week	2.42 (1.47)	3 (2–3)	2 (1.15)	2 (2–3)	0.095	3.55 (1.73)	4 (2.5–5)	3 (1.45)	3 (2–4)	0.188	0.022
4th week	2.19 (1.36)	3 (2–3)	2.56 (1.45)	3 (2–3)	0.432	3.1 (1.52)	3 (2–4)	3.89 (1.49)	4 (3–5)	0.211	0.046
5th week	2 (1.3)	2 (1–3)	2.8 (1.47)	3 (2–4)	0.022	2.65 (1.42)	3 (2–4)	3.89 (1.33)	4 (3–5)	0.015	0.121
6th week	1.73 (1.34)	2 (0–3)	2.72 (1.43)	3 (2–3)	0.008	2.6 (1.6)	3 (1.5–4)	4 (1.53)	4 (3–5)	0.019	0.056
7th week	1.5 (1.36)	2 (0–2)	2.64 (1.5)	3 (2–4)	0.007	2.3 (1.53)	2.5 (1.5–3)	4.32 (1.45)	5 (3–5)	<0.001	0.069
8th week	1.42 (1.36)	1.5 (0–2)	2.64 (1.5)	3 (2–4)	0.005	2.15 (1.6)	2 (0.5–3)	4.21 (1.58)	4 (3–5)	0.001	0.120
Total number of days consuming at least 5 drinks	16.42 (10.42)	19 (13–24)	20.32 (10.95)	22 (16–27)	0.155	24.4 (11.38)	25.5 (17–34.5)	30.53 (11.09)	30 (24–38)	0.143	0.024
Mean daily quantity of alcohol (1st- 4th week)	2.88 (1.8)	3 (3–4)	3.32 (1.31)	3 (3–4)	0.588	4.3 (1.66)	4 (3–5.5)	4.89 (1.29)	5 (4–6)	0.244	0.011
Mean daily quantity of alcohol (5th- 8th week)	1.58 (1.17)	2 (1–2)	3.24 (1.16)	3 (3–4)	<0.001	2.75 (1.45)	3 (2–3.5)	4.79 (1.23)	5 (4–5)	<0.001	0.004

*p*^1^—value for comparison between exercise and control group (Mann–Whitney test). *p*^2^—value for comparison between buprenorphine exercise group and methadone exercise group (Mann–Whitney test).

**Table 5:** Dosage of medication for opioid use disorders used by participants during the 8-week experimental period.

	Buprenorphine				*p* ^1^	Methadone				*p* ^1^	*p* ^2^
	Exercise Group		Control Group			Exercise Group		Control Group			
Substitute Dosage (mg/Week)	Mean (SD)	Median (IQR)	Mean (SD)	Median (IQR)		Mean (SD)	Median (IQR)	Mean (SD)	Median (IQR)		
Program onset	10.38 (1.5)	10 (10–12)	10 (1.41)	10 (10–10)	0.333	88.5 (21.59)	90 (70–105)	84.21 (18.05)	90 (60–100)	0.627	<0.001
1st week	10.38 (1.5)	10 (10–12)	10 (1.41)	10 (10–10)	0.333	88.25 (21.23)	90 (70–105)	84.21 (18.05)	90 (60–100)	0.627	<0.001
2nd week	10.38 (1.5)	10 (10–12)	10 (1.41)	10 (10–10)	0.333	88.25 (21.23)	90 (70–105)	83.68 (17.63)	90 (60–95)	0.587	<0.001
3rd week	10.23 (1.53)	10 (10–12)	9.76 (1.56)	10 (8–10)	0.278	87 (20.74)	87.5 (70–102.5)	83.68 (17.63)	90 (60–95)	0.732	<0.001
4th week	9.46 (1.56)	10 (8–10)	9.76 (1.56)	10 (8–10)	0.564	85 (20.84)	85 (67.5–102.5)	83.68 (17.63)	90 (60–95)	1.000	<0.001
5th week	9.38 (1.68)	10 (8–10)	9.76 (1.56)	10 (8–10)	0.506	82 (20.99)	82.5 (65–97.5)	84.21 (18.05)	90 (60–100)	0.590	<0.001
6th week	9 (1.72)	9 (8–10)	9.76 (1.56)	10 (8–10)	0.138	79.25 (20.79)	80 (62.5–95)	83.68 (18.84)	90 (60–100)	0.453	<0.001
7th week	8.31 (1.76)	8 (8–10)	9.6 (1.41)	10 (8–10)	0.007	75.75 (21.54)	75 (60–92.5)	82.11 (19.03)	85 (60–100)	0.257	<0.001
8th week	7.92 (1.92)	8 (6–8)	8.96 (1.31)	8 (8–10)	0.022	73.75 (20.96)	70 (60–90)	81.58 (18.86)	85 (60–100)	0.198	<0.001
Total substitute dosage (mg)	75.08 (12.04)	75 (66–84)	77.6 (11.25)	78 (68–80)	0.636	665.53 (167.61)	665 (525–830)	666.84 (145.27)	710 (480–785)	0.918	<0.001

*p*^1^—value for comparison between exercise and control group (Mann–Whitney test). *p*^2^—value for comparison between buprenorphine exercise group and methadone exercise group (Mann–Whitney test).

**Table 6:** Participants’ work and education days during the 8-week experimental period.

	Buprenorphine				*p* ^1^	Methadone				*p* ^1^	*p* ^2^
	Exercise Group		Control Group			Exercise Group		Control Group			
	Mean (SD)	Median	Mean (SD)	Median		Mean (SD)	Median	Mean (SD)	Median		
**Working days**											
Week 1	2.5 (2.18)	2 (0–5)	2.24 (1.96)	2 (0–4)	0.693	1.45 (1.79)	0 (0–3)	1.37 (1.8)	0 (0–3)	0.864	0.080
Week 2	2.5 (2.18)	2 (0–5)	2.24 (1.96)	2 (0–4)	0.693	1.45 (1.79)	0 (0–3)	1.37 (1.8)	0 (0–3)	0.864	0.080
Week 3	2.5 (2.18)	2 (0–5)	2.24 (1.96)	2 (0–4)	0.693	1.45 (1.79)	0 (0–3)	1.37 (1.8)	0 (0–3)	0.864	0.080
Week 4	2.77 (2.25)	4 (0–5)	2.24 (1.96)	2 (0–4)	0.426	1.7 (2.03)	0 (0–4)	1.37 (1.8)	0 (0–3)	0.608	0.068
Week 5	3.42 (1.9)	4 (2–5)	2.56 (1.89)	2 (2–4)	0.110	2.25 (2)	3 (0–4)	1.68 (1.8)	2 (0–3)	0.286	**0.025**
Week 6	3.46 (1.92)	4 (2–5)	2.56 (1.89)	2 (2–4)	0.090	2.3 (2.05)	3 (0–4)	1.68 (1.8)	2 (0–3)	0.273	**0.034**
Week 7	3.5 (1.92)	4 (2–5)	2.56 (1.89)	2 (2–4)	0.082	2.45 (2.11)	3.5 (0–4)	1.68 (1.8)	2 (0–3)	0.164	**0.043**
Week 8	3.5 (1.92)	4 (2–5)	2.72 (1.74)	2 (2–4)	0.109	2.5 (2.14)	4 (0–4)	1.68 (1.8)	2 (0–3)	0.139	**0.050**
**Total days** **of working**	24.15 (15.66)	26 (8–40)	19.36 (14.58)	16 (16–32)	0.263	15.55 (14.84)	14.5 (0–26)	12.21 (13.89)	12 (0–24)	0.378	0.056
	**Buprenorphine**				***p* ^1^**	**Methadone**				***p* ^1^**	***p* ^2^**
	**Exercise Group**		**Control Group**			**Exercise Group**		**Control Group**			
	**Mean (SD)**	**Median**	**Mean (SD)**	**Median**		**Mean (SD)**	**Median**	**Mean (SD)**	**Median**		
**Education Days**											
Week 1	1.65 (1.74)	2 (0–2)	1.56 (1.66)	1 (0–2)	0.905	1.05 (1.5)	0 (0–2)	1.05 (1.18)	0 (0–2)	0.727	0.211
Week 2	1.65 (1.74)	2 (0–2)	1.48 (1.66)	1 (0–2)	0.752	1.15 (1.5)	0 (0–2)	1.05 (1.18)	0 (0–2)	0.937	0.321
Week 3	1.77 (1.86)	2 (0–2)	1.56 (1.66)	1 (0–2)	0.781	1.2 (1.54)	0 (0–2)	1.05 (1.18)	0 (0–2)	0.950	0.320
Week 4	1.81 (1.92)	2 (0–2)	1.56 (1.66)	1 (0–2)	0.751	1.25 (1.62)	0 (0–2)	1.05 (1.18)	0 (0–2)	0.925	0.314
Week 5	2.08 (1.98)	2 (0–4)	1.88 (1.72)	2 (0–3)	0.785	1.55 (1.88)	1 (0–2)	1.05 (1.18)	0 (0–2)	0.600	0.360
Week 6	2.31 (2.02)	2 (0–4)	1.88 (1.72)	2 (0–3)	0.461	1.55 (1.88)	1 (0–2)	1.05 (1.18)	0 (0–2)	0.600	0.196
Week 6	2.38 (2.04)	2 (0–4)	1.88 (1.72)	2 (0–3)	0.383	1.6 (1.9)	1 (0–2.5)	1.05 (1.18)	0 (0–2)	0.509	0.197
Week 8	2.38 (2.04)	2 (0–4)	1.88 (1.72)	2 (0–3)	0.383	1.65 (1.93)	1 (0–3)	1.05 (1.18)	0 (0–2)	0.426	0.228
**Total days** **of education**	16.04 (14.7)	16 (0–24)	13.68 (13.1)	8 (0–23)	0.610	11 (13.3)	4 (0–17.5)	8.42 (9.42)	0 (0–16)	0.626	0.227

*p*^1^—value for comparison between exercise and control group (Mann–Whitney test). *p*^2^—value for comparison between buprenorphine exercise group and methadone exercise group (Mann–Whitney test).

## Data Availability

All data included in this study are available upon request by contact with the corresponding author.
